# Dialysis More Available Than Patient Education in Counties With High Diabetes Prevalence

**DOI:** 10.5888/pcd21.240052

**Published:** 2024-08-15

**Authors:** Janice C. Probst, Nicholas Yell, Gabriel A. Benavidez, Mary Katherine McNatt, Teri Browne, Laura Herbert, Whitney E. Zahnd, Elizabeth Crouch

**Affiliations:** 1 Rural and Minority Health Research Center, Arnold School of Public Health, University of South Carolina, Columbia; 2 Department of Public Health, Baylor University, Waco, Texas; 3 Department of Public Health, A.T. Still University College of Graduate Health Studies, Kirksville, Missouri; 4 College of Social Work, University of South Carolina, Columbia; 5 College of Nursing, University of South Carolina, Columbia; 6 Department of Health Management and Policy, RUPRI Center for Rural Health Policy Analysis, University of Iowa, Iowa City

## Abstract

**Introduction:**

Poorly controlled diabetes is a principal cause of end stage renal disease (ESRD), generating an estimated 44% of new cases. Diabetes self-management education and support (DSMES) has been documented to reduce adverse outcomes such as ESRD. Helping patients better manage their condition could ultimately reduce ESRD prevalence.

**Methods:**

We compared the county-level availability of DSMES and dialysis as of November 2022 sorted by the estimated prevalence of diabetes among residents aged 18 years or older. The locations of DSMES programs and ESRD dialysis facilities were obtained from 2 professional organizations and the Centers for Medicare & Medicade Services. Estimated diabetes prevalence was obtained from the Centers for Disease Control and Prevention’s PLACES data set. Counties were considered to have high diabetes prevalence if they fell into the top quartile for diabetes prevalence in 2019 (≥14.4% of adults). Analyses were conducted in 2023.

**Results:**

DSMES was available in 41.0% of counties but in only 20.7% of counties with high diabetes prevalence versus 47.9% of low prevalence counties. Dialysis facilities were present in 59.2% of all counties, in 52.8% of all high diabetes prevalence counties, and in 61.4% of other counties. DSMES availability was linked to the presence of a hospital in the county, with only 6.3% of counties without a hospital offering the service.

**Implications:**

DSMES could play a role in reducing the prevalence of ESRD. Public health professionals need to be aware of the differing levels of local availability of this service and work to develop partnerships to provide DSMES in high-prevalence areas not currently served.

**Summary**

**What is already known?**

Diabetes is a major contributor to the development of end stage renal disease (ESRD). Diabetes self-management education and support (DSMES), which could delay or avoid the onset of ESRD, is not used by all people with diabetes.

**What is added by this report?**

We compared the geographic availability, at the county level, of DSMES and dialysis, the principal treatment for ESRD. DSMES was less available than dialysis in counties falling in the top quartile for estimated diabetes prevalence.

**What are the implications for public health practice?**

Public health planners need to assess local availability of DSMES and partner to improve availability where needed.

## Introduction

Diabetes is a debilitating chronic disease and a major contributor to other chronic conditions (1). The disorder affected an estimated 11.6% of the US population in 2022, about 34 million people (2), and generated annual medical care costs estimated at $412.9 billion (3). In 2020 it was the eighth leading cause of death (4). Of particular importance, an estimated 44.0% of new diagnoses of end stage renal disease (ESRD) are diabetes-related (5,6). Both type 1 and type 2 diabetes contribute to the onset of kidney disease, principally through the adverse effects of excess glucose on blood vessels in the kidney (7).

Chronic kidney disease is estimated to affect 14% of US adults, with Black adults being particularly affected (18.8%) (8). When the disease progresses to the point where the kidneys no longer function — ESRD — the patient must receive dialysis or a kidney transplant to survive. As of 2021, 808,526 people in the US were living with ESRD.

ESRD care places burdens on the patient, the patient’s family, and the health care system. When the kidneys no longer function, waste must be cleared from the body by external devices. The most common treatment, hemodialysisis, is conducted at a dedicated facility 3 or 4 times a week, and sessions can interfere with work or other activities. The resources and stress associated with both the transplant process and hemodialysis affect family well-being (9). Finally, the cost of ESRD to the US health care system exceeded $50 billion in 2021 and made up about 6.8% of Medicare expenditures (8).

Improved diabetes care may help reduce the prevalence of kidney disease. The adverse effects of diabetes are reduced when people with the disorder successfully manage their condition. They must monitor their blood glucose levels, adhere to diet and exercise guidelines, and take appropriate medications. Because the complexity of diabetes management goes beyond typical instructions for outpatient care, diabetes self-management education and support (DSMES) programs that use standardized, evidence-based curricula have been developed (10,11). DSMES has been shown to improve glycemic control and reduce mortality among people with diabetes (12,13), and it is a covered service under Medicare. In addition, 43 states require DSMES coverage for private insurers and Medicaid (14). Despite these efforts to promote DSMES, patient participation remains low, with one estimate showing that slightly more than half of people with diabetes report receiving this education (15). Lack of DSMES providers at the local level may be one factor affecting patient participation (16).

Because diabetes is an important pathway to the development of ESRD, we sought to compare the relative geographic availability of DSMES and dialysis services. From a prevention viewpoint, ensuring that DSMES is available might serve to reduce the need for ESRD treatment over the long term. Areas that have only dialysis facilities for treatment of this major diabetes outcome but lack DSMES for prevention require public health attention. We used a combination of data sources to identify counties that have a high need for DSMES, based on estimated diabetes prevalence, and assessed the services available in these counties.

## Methods

We conducted a cross-sectional analysis in 2023 of the availability of DSMES and dialysis facilities in US counties (N = 3,141). Data from the multiple sources that follow were linked for the analysis.

### Diabetes self-management education

We obtained addresses of all accredited DSMES providers as of December 2022 from the American Diabetes Association and the Association of Diabetes Care and Education Specialists. All locations offering DSMES, both main and branch locations, were included in these lists. This information is also publicly available on the respective organizations’ websites. Addresses were geocoded to the county level by using the US Housing and Urban Development’s (HUD’s) USPS ZIP Code Crosswalk Files (17). Because zip codes can overlap county boundaries, the HUD algorithm uses the physical location of most businesses and residences to assign a zip code to a county. Assessment suggests that the rate of misassignment at the county level is minimal (18). Online accredited programs and services of the Veterans Administration and Department of Defense were not included. 

### Dialysis

We downloaded a list of Medicare-certified dialysis facilities as of November 16, 2022, from the Centers for Medicare & Medicaid Services (CMS) website (19). Addresses were geocoded to the county level. Dialysis is not the only treatment for kidney failure; a kidney transplant is an alternative approach. However, the presence of a dialysis facility in the county suggests access to clinicians such as nephrologists who could refer patients for transplant (20).

### Estimated population with diabetes

The CDC PLACES (Population Level Analysis and Community Estimates) data set provided estimates for the proportion of the adult population with diabetes (21). PLACES prevalence values are based on self-report of diabetes in the 2019 Behavioral Risk Factor Surveillance System survey, with statistical modeling used to create county-level estimates. The PLACES data set did not include information for 2 of the 3,143 US county equivalents, the Chugach Census Area and the Copper River Census Area in Alaska; thus, the total counties studied was 3,141. Counties were designated as high prevalence if the estimated proportion of the adult population with diabetes was in the highest quartile across all counties (≥14.4%, n = 790, range 14.4% – 25.9%) versus all lower quartiles (≤14.3%, n = 2,351, range 7.3% – 14.3%).

### County characteristics

Characteristics such as population size, demographics, areas with shortages of health care clinicians per Health Professional Shortage Area (HPSA) guidelines, and access to health care resources such as a hospital or a federally qualified health center (FQHC), were drawn from the Robert Wood Johnson Foundation County Health Rankings data set, 2022 edition (22,23). Definitions for rurality were obtained from the Economic Research Service of the US Department of Agriculture and are based on Urban Influence Codes (24).

### Analysis

We used χ^2^ cross-sectional analyses to test for categorical differences and the Mann–Whitney test to examine medians. Because the study was primarily descriptive, we did not conduct multivariable analyses. All analyses were conducted in Stata version 18 (StataCorp LLC) with an α level of .05. We used ArcGIS version 10 (Esri) to produce all maps. Our research was deemed exempt by the institutional review board of the University of South Carolina.

## Results

### Estimated diabetes prevalence

The national estimated county-level prevalence of diabetes among adults ranged from a low of 6.2% to a high of 25.9%. Counties falling in the highest quartile for prevalence had an average adult prevalence of 16.2%, versus 11.6% in other counties.

The Southern region had the highest proportion of counties with high diabetes prevalence (47.5%) (Table 1). Overall, most counties with high diabetes prevalence were in the South (675 of 790 [85.4%] high-prevalence counties). High prevalence counties were more likely than their counterparts to be rural, to have entire or partial shortages of primary care clinicians, to be served by a federally qualified health center, and to lack a hospital (Table 1). Examining demographic characteristics, high prevalence counties contained relatively high proportions of non-White residents, uninsured adults, and child poverty households (Table 2). Of relevance for access to online DSME, the median proportion of households with broadband access in high-prevalence counties was 71.4%, versus 81.6% in low-prevalence counties (Table 2).

**Table 1:** Characteristics of US Counties (N = 3,141)^ a^, By Estimated Diabetes Prevalence^b^ Among Adults Aged 18 Years or Older, November 2022

Characteristic	Total, n (%)	High estimated diabetes prevalence, n (%)	Low estimated diabetes prevalence, n (%)	*P* value^c^
**All counties**	3,141 (100.0)	790 (25.2)	2,351 (74.9)	NA
**Service availability, %**				
DSMES	1,289 (41.0)	164 (12.7)	1,125 (87.3)	<.001
Dialysis	1,860 (59.2)	417 (22.4)	1,443 (77.6)	
**Rurality, %**				
Urban	1,166 (37.1)	150 (12.7)	1,016 (87.1)	<.001
Rural (all)	1,975 (62.9)	640 (32.4)	1,335 (67.6)	
Rural micropolitan	641 (20.4)	147 (22.9)	494 (77.1)	
Rural noncore	1,334 (42.5)	493 (37.0)	841 (63.0)	
**Census region, %**				
Northeast	217 (6.9)	2 (0.9)	215 (99.1)	<.001
Midwest	1,055 (33.6)	69 (6.5)	986 (93.5)	
South	1,422 (45.3)	675 (47.5)	747 (52.5)	
West	447 (14.2)	44 (9.9)	403 (90.2)	
**Counties having HPSA status or health care resource, %**				
Whole county HPSA status	23.2	40.1	17.4	<.001
FQHC	65.5	77.3	61.6	<.001
RHC	74.2	86.5	70.1	<.001

Abbreviations: DSMES, diabetes self-management education and support; FQHC, federally qualified health center; HPSA, health professional shortage area; RHC, rural health center.

a The PLACES data set does not include information for 2 county equivalents in Alaska, the Chugach Census Area and the Copper River Census Area.

b High prevalence = ≥14.4%; low prevalence = ≤14.3%.

c Calculated by using χ^2 ^or Fisher Exact Test, as appropriate.

**Table 2:** Demographic Characteristics of US Counties (N = 3,141)^a^, by Estimated Diabetes Prevalence^b^, November 2022

Characteristic	Total, median	High estimated diabetes prevalence (n = 790)	Low estimated diabetes (n = 2,351)	*P* value^c^
**Total county population**				
**Median number of residents**	25,658	16,714	33,825	<.001
**Demographic characteristic, percentage of population**				
Race or ethnicity				
Asian/Pacific Islander	0.9	0.6	1.0	<.001
Hispanic	4.7	3.6	5.0	<.001
Non-Hispanic American Indian/Alaska Native	0.7	0.6	0.7	.04
Non-Hispanic Black	2.3	9.7	1.8	<.001
Non-Hispanic White	82.8	62.6	85.6	<.001
Age, ≥65 y	19.8	20.8	19.5	<.001
**Resources, median percentage **				
Population <18 y with income below federal poverty level	17.6	27.5	15.1	<.001
Uninsured adults aged 18–64 y	12.8	17.3	11.5	<.001
Unemployment among people in the workforce aged ≥16 y	6.5	7.3	6.3	<.001
Education, high school (% 9^th^ graders who graduated in 4 years)	88.8	82.6	90.4	<.001
Not English fluent	0.7	0.6	0.8	<.001
Broadband access (household)	79.6	71.4	81.6	<.001

a The PLACES data set does not include information for 2 county equivalents in Alaska, the Chugach Census Area and the Copper River Census Area.

b High prevalence = ≥14.4%; low prevalence = ≤14.3%. Counties are sorted by prevalence of diabetes among adults, but characteristics pertain to the whole population.

c Calculated by Mann–Whitney test.

### DSMES availability

**Figure 1:**
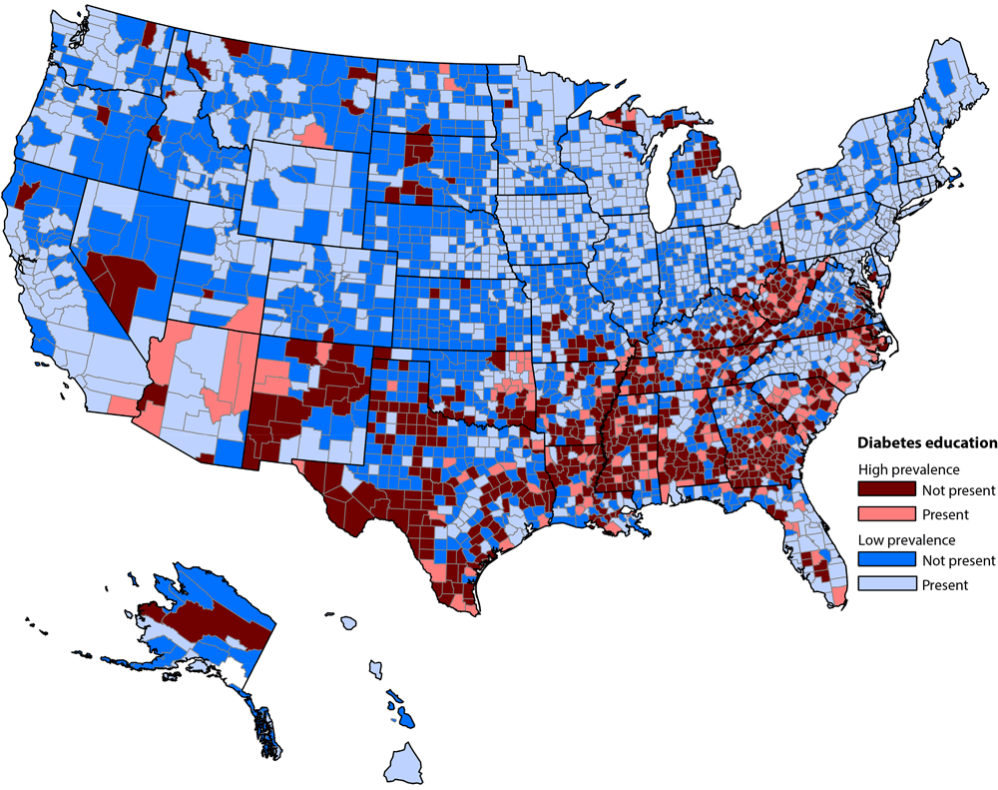
Availability of diabetes self-management education and support (DSMES) and county (N = 3,141) diabetes prevalence (top quartile [≥14.4%] versus all lower quartiles [≤14.3%], 2019 estimates) (21). The PLACES data set does not include information for 2 county equivalents in Alaska, the Chugach Census Area and the Copper River Census Area. Source: Centers for Disease Control and Prevention. PLACES: Local Data for Better Health (21).

Across the US, 41.0% of all counties contained at least 1 location where DSMES was offered (1,289 counties) (Table 1). However, DSMES was available in only 20.7% of counties with high diabetes prevalence, versus 47.9% of low-prevalence counties (Figure 1). Of note, only 30.1% of rural counties had an in-county DSMES program (Table 3). DSMES availability was linked to the presence of health care resources in the county. Counties designated as whole county Health Professional Shortage Areas for primary care were unlikely to have locally available DSMES (Table 3).

**Table 3:** Availability of DSMES and Dialysis, by County Characteristics (N = 3,141)^a^, November 2022

Characteristic	Total	Resource combinations within the county				
		DSMES & dialysis, n (%)	DSMES only, n (%)	Dialysis only, n (%)	Neither resource, n (%)	*P* value^b^
All counties	3,141	1,085 (34.5)	204 (6.5)	775 (24.7)	1,077 (34.3)	NA
**Diabetes prevalence**						
Top quartile (≥14.4%)	790	129 (16.3)	35 (4.4)	288 (36.5)	338 (42.8)	<.001
Bottom 3 quartiles (≤14.3%)	2,351	956 (40.7)	169 (7.2)	487 (20.7)	739 (31.4)	
**Rurality**						
Urban	1,166	663 (56.9)	32 (2.7)	266 (22.8)	205 (17.6)	<.001
Rural (all)	1,975	422 (21.4)	172 (8.7)	509 (25.8)	872 (44.1)	
Rural micropolitan	641	281 (43.8)	29 (4.5)	235 (36.7)	96 (15.0)	
Rural noncore	1,334	141 (10.6)	143 (10.7)	274 (20.5)	776 (58.2)	
**Census region**						
Northeast	217	146 (67.3)	8 (3.7)	44 (20.3)	19 (8.8)	<.001
Midwest	1,055	362 (34.3)	110 (10.4)	181 (17.2)	402 (38.1)	
South	1,422	421 (29.6)	48 (3.4)	483 (34.0)	470 (33.1)	
West	447	156 (34.9)	38 (8.5)	67 (15.0)	186 (41.6)	
**Health care resources**						
Whole County HPSA						
Yes	727	56 (7.7)	42 (5.8)	187 (25.7)	442 (60.8)	<.001
No	2,414	1,029 (42.6)	162 (6.7)	588 (24.4)	635 (26.3)	
FQHC in county						
Yes	2,058	889 (43.2)	89 (4.3)	562 (27.3)	518 (25.2)	<.001
No	1,083	196 (18.1)	115 (10.6)	213 (19.7)	559 (51.6)	
RHC in county						
Yes	NA	638 (27.4)	172 (7.4)	623 (26.7)	899 (38.6)	<.001
No	NA	447 (55.3)	32 (4.0)	152 (18.80)	178 (22.0)	
Hospital in county						
Yes	2,466	1,067 (43.3)	180 (7.3)	667 (27.1)	552 (22.4)	<.001
No	675	18 (2.7)	24 (3.6)	108 (16.0)	525 (77.8)	

Abbreviations: DSMES, diabetes self-management education and support; FQHC, federally qualified health center; HPSA, Health Professional Shortage Area; NA, not applicable; RHC, rural health clinic.

a The PLACES data set does not include information for 2 county equivalents in Alaska, the Chugach Census Area and the Copper River Census Area.

b Calculated by χ^2 ^or Fisher Exact Test, as appropriate.

Examination of the infrastructure associated with in-county DSMES showed that counties that had both a hospital and an FQHC were most likely to also contain DSMES (57.1%), followed by counties with a hospital but not an FQHC (37.2%) (Table 4). Only 272 of 3,141 counties studied (8.7%) did not have at least 1 of these facilities. However, even among counties with both a hospital and an FQHC, 42.9% lacked DSMES.

**Table 4:** DSMES Availability in US Counties (N = 3,141)^a^, by the Presence of an FQHC or a Hospital in the County

Availability	Total	Type of health care service				
		Both hospital & FQHC, n (%)	FQHC only, n (%)	Hospital only, n (%)	Neither, n (%)	*P* value^b^
**Counties health care services**	3,141	1,655 (52.7)	403 (12.8)	811 (25.8)	272 (8.7)	NA
**DSMES in county**						
Yes	1,289	945 (57.1)	33 (8.2)	302 (37.2)	9 (3.3)	<.001
No	1,852	710 (42.9)	370 (91.8)	509 (62.8)	263 (96.7)	

Abbreviations: DSMES, diabetes self-management education services; FQHC, federally qualified health center.

a The PLACES data set does not include information for 2 county equivalents in Alaska, the Chugach Census Area and the Copper River Census Area.

b Calculated by χ^2^.

### Dialysis availability

**Figure 2:**
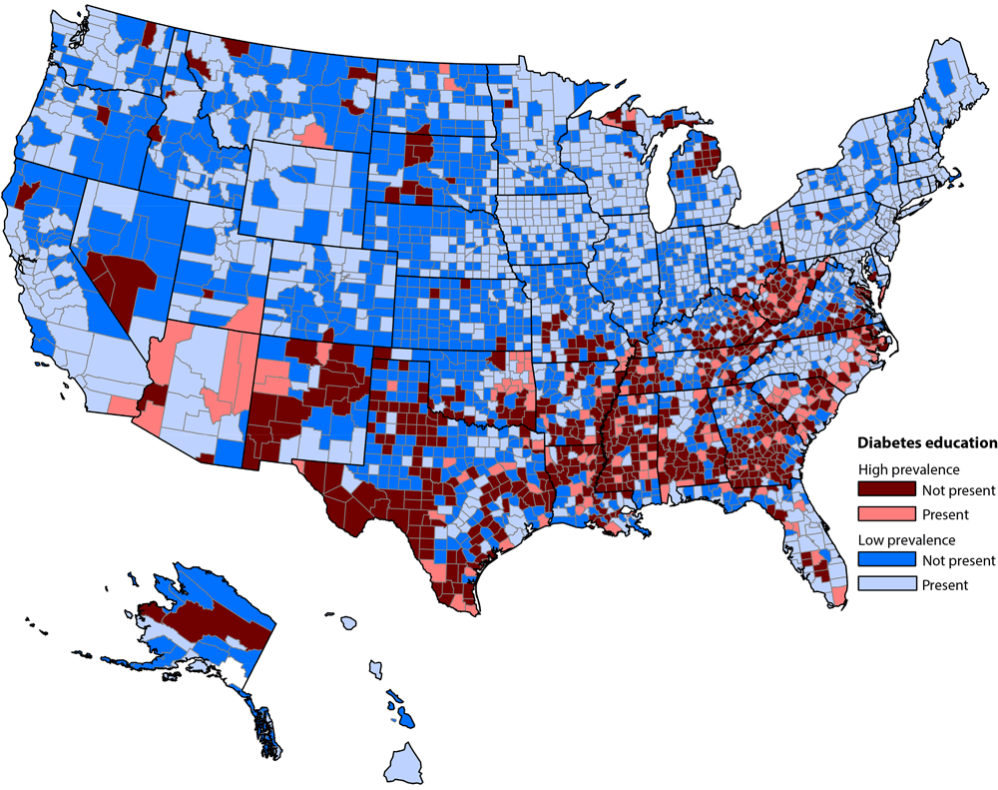
Dialysis availability and county diabetes prevalence (top quartile [≥14.4%] versus all lower quartiles [≤14.3%]), 2019 estimates) (21). DSMES, either alone or with dialysis, was available in 417 (52.8%) counties. The PLACES data set does not include information for 2 county equivalents in Alaska, the Chugach Census Area and the Copper River Census Area. Source: Centers for Disease Control and Prevention. PLACES: Local Data for Better Health (21).

Dialysis services were more broadly available than DSMES, with at least 1 facility present in 59.2% of all counties (Table 1). Within the 790 counties with high diabetes prevalence, dialysis, either alone (36.5%) or with DSMES (16.3%) (Table 3), was available in 417 counties (52.8%) (Figure 2). Having a dialysis facility but no DSMES, that is, treatment facilities but no prevention activities, was more common in high-prevalence than low-prevalence counties, 36.5% versus 20.7%, respectively.

## Discussion

Reflecting the inverse care law (25), DSMES was less available in counties in the top quartile for estimated diabetes prevalence than in other counties. Paradoxically, a larger proportion of high-prevalence counties had the resources to treat ESRD (52.8%) than to provide education that might lower its prevalence (20.7%). Nearly all counties studied contained either a hospital or an FQHC; only a small fraction had neither (8.7%). Nonetheless, many counties that housed these facilities did not have a local DSMES program. Even when a county had both types of facilities, 42.9% did not have DSMES.

Public health practitioners could partner with hospitals and FQHCs to expand DSMES availability. Nonprofit hospitals are required to provide community benefit services to maintain their tax-exempt status; however, most funding for community benefit goes to charity care. Only a small portion is allocated for education (26). 

Several potential barriers need to be addressed for partnerships to be successful at expanding DSMES availability. Unlike dialysis, DSMES does not have a guaranteed funder, nor is it a substantial revenue source. Since 1973, Medicare has been the guaranteed payor for all ESRD care, including dialysis (27,28). Dialysis generates considerable income for providers. Its annual cost in 2021 was estimated at approximately $99,000 per patient for hemodialysis and $87,000 for peritoneal dialysis (8). DSMES, on the other hand, is billed at roughly $56 per 30-minute individual session, and about $16 per person for group sessions (2022 national average rates) (29). In addition, clinicians cannot bill Medicare for DSMES on the same day as a visit for other purposes, making it difficult to bundle services to promote patient engagement. Not only does this reduce potential income for the clinician, but it may also decrease patient participation. Of note for underserved populations, Medicare restricts the degree to which FQHCs may bill for DSMES (30). Finally, because DSMES providers can bill Medicare for only 10 sessions during a patient’s first year of a diabetes diagnosis or first year on Medicare, and 2 hours per year thereafter, DSMES is not a substantial revenue-generating service.

Another factor affecting availability of DSMES is that a diabetes education provider must meet certain requirements to be certified (10). DSMES educators must document their education, the curriculum they will use for patient education, quality control activities, and other features of the education program. Public health agencies may need to partner with local hospitals or other health care professionals to find ways to subsidize the process of obtaining DSMES certification and the cost of providing the service to patients. Finally, multiple barriers restrict use of DSMES, some of which could be reduced by policy changes. For DSMES to be billable, the patient’s health care provider must provide a written or electronic order for that service (31). Thus, communication with clinicians is necessary to make DSMES programs available to patients, including online, virtual, or in-person courses. Adjustments to patient flow or prompts in the electronic health record may be needed for referrals to take place. Allowing patients to seek out reimbursable DSMES on their own if they had not received it in the past might reduce this barrier. In addition, the Centers for Medicare & Medicaid Services restricts locations where in-person DSMES may be offered, requiring that for DSMES to be billable, it must take place in a medical setting such as a hospital outpatient department or private clinical practice. The only allowable community location is a pharmacy. Permitting the use of other spaces for DSMES, such as schools, libraries, or faith-based organizations, would expand options for rural communities that lack health care facilities.

Certified online DSMES could ameliorate the lack of local, in-person education programs. However, we found that broadband access was lower in counties with high diabetes prevalence than in other counties, and broadband access has consistently been lower in rural areas (32,33). The Bipartisan Infrastructure Law, which allocated $65 billion to extending internet access nationally, provided funding both for installation of broadband technology in unserved communities and for subsidies to allow low-income households to pay for services (34). As of this writing, however, the $30 per month broadband subsidy for low-income households has not been renewed, raising concerns that improvements in broadband access will be reversed (35). In addition, although the proportion of households lacking broadband will probably decline over the next decade, public health planners will still have to address the issues of health and internet literacy among populations learning to use these tools.

When DSMES is available locally, it may not be within financial reach of all patients. Although Medicare and Medicaid, as well as private insurers in nearly all states, treat DSMES as a reimbursable service, they do not waive patient financial responsibility. Low-income and uninsured people are less likely to report having had DSMES than their counterparts, suggesting that cost may be a barrier (15). Similarly, uptake of DSMES among Medicare fee-for-service beneficiaries is not optimal (36). The federal government has moved to cap the cost of insulin for Medicare beneficiaries (37), with possible spillover effects on private insurers (38). Similar initiatives or targeted subsidies could address reducing the cost of DSMES for patients. Incentives might also be needed to prompt clinicians to offer this low-revenue service. These relatively low-cost investments in DSMES may be an effective strategy in the long run for reducing the prevalence of ESRD.

### Limitations

Our study had several limitations. First, it was ecological, examining counties. Relatedly, the county measure may include a small number of facilities incorrectly assigned by the US Department of Housing and Urban Development’s Zip Code Crosswalk Files (18). Second, our measure of need, diabetes prevalence among adults, is based on model-based estimates and thus subject to any limitations present in the modeling process. Third, we may not have captured all diabetes education. Although insurers will pay only for DSMES provided by certified programs, organizations may elect to offer diabetes education informally. However, the extent to which providers offer services that cannot be billed is likely to be low; in addition, the quality of such education could not be documented. CDC recommends that DSMES providers become certified (39). Next, our analysis did not include Indian Health Service DSMES programs unless they were CMS certified. The Indian Health Service both promotes diabetes education and supports the Special Diabetes Program for Indians, which reported 301 sites in 2020 (40). Finally, although the benefits of DSMES participation have been documented, whether geographic availability of DSMES is linked to patient participation or overall health outcomes is unknown.

### Conclusion

We recommend that state and local public health departments assess the availability of DSMES in their service areas to determine whether the needs of people with diabetes are adequately addressed. Expanding the availability of DSMES is essential for reducing the adverse sequelae of this disease. Three options may improve access to DSMES: prompting additional providers to offer the service, expanded availability of online DSMES, and reducing associated patient costs, regardless of mode. Improved access could reduce both the immediate and long-term health effects and costs of diabetes and the incidence of ESRD, both for patients and for the health care system as a whole.
